# Bark-inhabiting fungal communities of European chestnut undergo substantial alteration by canker formation following chestnut blight infection

**DOI:** 10.3389/fmicb.2023.1052031

**Published:** 2023-01-26

**Authors:** Clovis Douanla-Meli, Julia Moll

**Affiliations:** ^1^Julius Kühn Institute (JKI) – Federal Research Centre for Cultivated Plants, Institute for National and International Plant Health, Quedlinburg, Germany; ^2^Department of Soil Ecology, Helmholtz Centre for Environmental Research-UFZ, Halle (Saale), Germany

**Keywords:** fungal interactions, *Cryphonectria parasitica*, *Gnomoniopsis smithogilvyi*, *Castanea sativa*, amplicon sequencing, mycobiome, metabarcoding

## Abstract

**Background:**

Chestnut forests are severely threatened by chestnut blight caused by the fungal pathogen *Cryphonectria parasitica* and the infected trees exhibit bark canker in the later stage of the disease. European chestnut (*Castanea sativa*) is further infected by *Gnomoniopsis smithogilvyi*, another canker-causing fungal pathogen. We explored whether and how chestnut blight is reflected in bark-inhabiting fungal communities of European chestnut and also assessed the co-occurrence of *C. parasitica* and *G. smithogilvyi*.

**Materials and methods:**

We initially investigated the fungal communities of European chestnut bark tissues and further monitored changes in these fungal communities with regard to disease progression from infection to canker formation by analyzing bark samples from asymptomatic trees, asymptomatic trees with latent *C. parasitica infection*, and infected trees with canker tissues, using amplicon sequencing of the ITS2 region of rDNA.

**Results:**

The results showed that fungal community composition and diversity differed between the sample types. The fungal community composition was substantially reshaped by canker formation, whereas latent *C. parasitica infection* and more specifically pre-canker infection period per se had a weak effect. Fungal communities of canker samples was less diverse and more dissimilar to those of other sample types. *C. parasitica* dominated the mycobiome of canker samples, whereas *G. smithogilvyi* was found in only 9% of canker samples at very low abundances. However, *G. smithogilvyi* was a dominant fungus in the bark of healthy plants.

**Conclusion:**

This study highlights that canker formation is the principal driver of decreasing diversity and altered composition of the mycobiome in bark tissues of European chestnut infected by *C. parasitica* infection. It additionally emphasizes the scarce co-occurrence of *C. parasitica* and *G. smithogilvyi* on European chestnut.

## Introduction

Each living plant supports a certain diversity of fungi such as endophytes, or pathogens (Arnold and Lutzoni, 2007; Rodriguez et al., 2009; Griffin and Carson, 2018). This fungal community can vary spatially and temporally owing to the influence of biotic and abiotic factors (Linaldeddu et al., 2010; Santos-Medellín et al., 2017; Carper, 2018; Kolp et al., 2020). Changes in plant fitness and plant tissue alteration can create new local microhabitats, thereby influencing mycobiome composition and diversity (Carper, 2018; Raza et al., 2019). While some species may adapt to new microhabitat conditions, others disappear or are outcompeted (Lappalainen et al., 1999; Douanla-Meli et al., 2013; Kolp et al., 2018). Physiochemical changes in plant tissues due to fungal diseases can facilitate tissue colonization by the disease-causing agent but also out-competition of the co-occurring endophytes or other species with pathogenic potential (Busby et al., 2016; Ćurković-Perica et al., 2017; Luo et al., 2019; Raza et al., 2019; Kolp et al., 2020).

European chestnut (*Castanea sativa* Mill.) is extensively cultivated for nuts and timber (Anagnostakis, 1987) and is widely distributed in Europe and Western Asia representing an important component of broadleaf forests (Conedera et al., 2016). European chestnut forests are prone to biotic disturbances, such as canker diseases caused by the fungal pathogens *Cryphonectria parasitica* and *Gnomoniopsis smithogilvyi*, two *Ascomycetes* of the order *Diaporthales*. Native to Eastern Asia, *C. parasitica* is a major threat to *Castanea* spp. worldwide (Pasche et al., 2016; Rigling and Prospero, 2018). *Gnomoniopsis smithogilvyi* is mainly a pathogen on chestnut fruits, but endophytically colonizes stems and branches (Vettraino et al., 2011; Pasche et al., 2016; Lewis et al., 2017). Some currently unknown circumstances may trigger its transition from endophyte to a canker pathogen, causing symptoms similar to those of chestnut blight (Dar and Rai, 2015; Pasche et al., 2016). A specific symptom of chestnut blight is blushing and cracking of the bark at the infection point on the trunk and branches (Supplementary Figure S1A), which often becomes populated with yellow-orange to red fruiting bodies (Supplementary Figures S1B,D; Pasche et al., 2016; Rigling and Prospero, 2018). The fungus grows in the inner bark (phloem) and cambium tissue. Cankers then develop following tree response and cover the destroyed bark tissues, which turn brown or become dark (Supplementary Figure S1C; Pasche et al., 2016; Kolp et al., 2020). These changes in the texture, structure and chemistry of the bark tissue imply modification of the local microhabitat, which may affect the composition and richness of the mycobiome (Kolp et al., 2018, 2020). Shifts in fungal community structure can in turn influence chestnut blight severity (Kolp et al., 2020). To understand the relationship between *C. parasitica*, canker formation and European chestnut bark-inhabiting fungal community, it is essential to compare fungal communities associated with healthy and infected tissues.

The shift in the fungal community of chestnut canker has been relatively well examined and demonstrated to hold a key role in the destiny of canker (Prospero and Rigling, 2016; Ćurković-Perica et al., 2017; Kolp et al., 2020). Specially, the ongoing antagonistic interactions among co-occurring fungi, e.g., healthy and virulent *C. parasitica*, *C. parasitica* infected with a hypovirus and other fungal species, were considered to represent a determinant factor influencing the canker fungal diversity, which in turn affects the canker expansion (Akilli et al., 2011; Ćurković-Perica et al., 2017; Kolp et al., 2020). However, knowledge of the overall succession of fungal communities in *C. parasitica*-infected bark tissues is limited. Considering that previous studies of chestnut fungal communities have focused primarily on cankers (Akilli et al., 2011; Kolp et al., 2020) the effects of initial infection by *C. parasitica,* as well as that of canker formation on chestnut mycobiome has not been explored. Filling this knowledge gap requires further investigation of the whole mycobiome in healthy bark tissues and its change from the time of infection by *C. parasitica* to the emergence of canker tissues in European chestnut. Previous studies have mainly used culture-based fungal community analyzes (Akilli et al., 2011; Kolp et al., 2018, 2020) with the drawback that many endophytic fungi are uncultivable (Qian et al., 2019). Currently, the use of culture-independent DNA-based amplicon sequencing (next generation sequencing) has broadened our knowledge of plant-associated fungal communities and is increasingly used to routinely access fungal communities (Taylor et al., 2014; Abdelfattah et al., 2015; Fort et al., 2016; Hugerth and Anderson, 2017). This technique will provide more complete picture of plant-associated fungal diversity and thus unmask fungal community patterns related to chestnut blight.

We took advantage of next-generation sequencing to examine fungal communities in the bark tissues of healthy and infected European chestnut. Important plantations of European chestnut are located around the Rhine Valley in southwestern Germany, where *C. parasitica* has been established since 1992, causing significant damage (Seemann and Unger, 1993; Peters et al., 2012, 2014). A previous study on infection progress (Wambsganß et al., 2015) and annual monitoring by the plant protection service localized some disease-free areas. We selected different sites in chestnut plantations with or without the disease and sampled bark tissues from asymptomatic and diseased trees. The selected samples were further analyzed by amplicon sequencing to explore fungal communities. The main objectives were (1) to assess the overall fungal communities of European chestnut bark tissues, (2) to compare fungal communities in healthy and *C. parasitica*-infected bark tissues to evaluate the impact of canker formation, and (3) to assess the occurrence of *G. smithogilvyi* and its co-occurrence with *C. parasitica*.

## Materials and methods

### Sampling of chestnut bark tissue

Sampling was performed in the states of Baden-Württemberg and Rhineland-Palatinate around the Rhine Valley located in southwestern Germany. The Rhine Valley with semi-continental climate is one of the rainiest and warmest regions in Germany. Average annual precipitation ranges between 500 and 700 mm and average annual temperature ranges from 10.8°C to 11.2°C, with characteristically dry summer weather conditions (Statista 2022). Local climatic conditions make this area unique to the extensive chestnut forests in Germany. Current plant diversity patterns include pure chestnut stands, mixed chestnut-oak and unmixed oak stands. Chestnut blight has quickly spread in this area leading to severe infection in chestnut plantations with only a few disease-free patches (Seemann and Unger, 1993; Peters et al., 2012, 2014). In this study, 12 sites with confirmed chestnut blight were selected in Baden-Württemberg, in the localities of Oberweier, Oftersheim, and Gernsbach. Four sites that remained disease-free were selected in Rhineland-Palatinate, located in Weyer in der Pfalz (Supplementary Figure S2). We sampled bark tissues from asymptomatic (putatively healthy) and symptomatic trees (showing infection with a developed bark canker). At sites with chestnut blight infection, samples were taken from healthy tissues of asymptomatic trees, healthy tissues of infected trees, and cankered tissues of infected trees. Healthy tissues from asymptomatic trees were sampled at sites free of chestnut blight.

Samples were taken independently and at different growth stages. Sampling was conducted twice, between November and December 2019 and 2021. We used random sampling, but selected trees with a distance of at least 5 m to each other. A 5-mm-diameter cork borer was used to collect the bark tissue down to the cambium. The cork borer was thoroughly washed and surface sterilized with 3% hypochlorite between each cutting to prevent cross-contamination among samples. Each sample consisted of two tissue pieces taken at a distance of 10 cm. From trees with canker, samples were taken at the center of the cankers and from healthy tissues at least 20 cm from the canker margin. Samples were sealed in sterile paper bags, and transported in a cooling box. In the laboratory, they were stored at +4°C until processing the following day. All samples were subjected to real-time PCR analysis (Chandelier et al., 2019) to detect *C. parasitica*. Thereafter, samples were grouped according to their health condition and negative or positive real-time PCR results into four categories: HTHT-nCp (healthy tissue from healthy trees), HTHT-Cp (healthy tissue from healthy trees with *C. parasitica*), HTIT-nCp (healthy tissue from infected trees without *C. parasitica*), and CTIT-Cp (canker tissue from infected trees with *C. parasitica*). All canker samples were positive for *C. parasitica* by real-time PCR.

### DNA extraction and real-time PCR amplification

Tissue samples (0.5 g) were collected from the inner bark (phloem) and cambium layers to avoid contamination by epiphytic fungi on the outer bark. For bark tissue grinding and homogenization, a mixer mill MM 200 (Retsch, Germany) was used, and three tungsten carbide balls of 3 mm diameter were included in each cup. DNA was extracted using a Nucleospin Plant II Mini Kit according to the manufacturer’s instructions. The DNA was quantified with a NanoDrop ND-1000 spectrophotometer (NanoDrop Technologies, Wilmington, DE, United States) and stored at −18°C. Amplification by TaqMan™-based real-time PCR assay (Chandelier et al., 2019) was carried out to check for the presence of *C. parasitica* in the samples. The assay was performed in duplicate using the primers Cp-F4 (GATACCCTTTGTGAACTTATAA) and Cp-R3 (GGGGAGAAGGAAGAAAATC) in combination with the probe Cp-S3 (FAM-TTTATCGTTGCCTCGGCGCTGA-BHQ1). PCR was carried out in 20 μl using the Maxima Probe/Rox qPCR master mix (Thermo Fisher Scientific, Germany) in a *qTOWER*^3^ Real-Time PCR thermal cycler (Analytik Jena, Germany), according to the cycling parameters described by Chandelier et al. (2019).

### Sequencing and bioinformatics analysis

The DNA from the samples was sent to Microsynth AG (Balgach, Switzerland) for amplicon sequencing. Primers fITS7 and ITS4, best suited for analyzing between-sample differences in fungal community composition (Ihrmark et al., 2012), were used to amplify the fungal internal transcribed spacer (ITS2) region (Schoch et al., 2012; Nilsson et al., 2019a). For paired-end sequencing, PCR libraries were created and sequenced on an Illumina MiSeq platform. Sequences were deposited in the European Nucleotide Archive (ENA) database under the project accession number PRJEB46887.

The Dadasnake pipeline, which includes the DADA2 workflow and several other bioinformatics tools, was used to process the sequencing data (Callahan et al., 2016; Weißbecker et al., 2020). Both primer sequences were cut using Cutadapt v1.18 (Martin, 2011). Quality filtering was performed using the default settings for fungal ITS sequencing (maxEE = 2, truncQ = 2, truncLen = 0). The remaining forward and reverse reads were merged with an overlap of 20 bp, one mismatch was allowed. Chimeras were removed based on the “consensus” method and the obtained ASVs (amplicon sequence variants) were taxonomically assigned using the Bayesian Classifier implemented in mothur (Schloss et al., 2009) against the UNITE database (version 8; Nilsson et al., 2019b). Thereafter, all ASVs assigned to the kingdom “fungi” were subjected to post-clustering at 97% sequence similarity using VSEARCH (Rognes et al., 2016) to consider intraspecific sequence variation of the fungal ITS region (Estensmo et al., 2021).

### Statistical analysis

All statistical analyzes were performed using R Version 4.0.2 (R Core Team, 2020) and the interface RStudio (Version, RStudio Inc., Boston, USA). Only samples comprising more than 5,000 sequences were considered for further analysis. This dataset was normalized by rarefying the lowest number of sequences per sample (5,543 sequences) to allow comparison between sample types using the package “phyloseq” (McMurdie and Holmes, 2013). Rarefaction curves were visualized using the “ggrare” function of the “ranacapa” package (Kandlikar et al., 2018). Taxonomic composition at phylum, class and genus levels was analyzed using the packages “phyloseq” and “microeco” (McMurdie and Holmes, 2013; Liu et al., 2021). Fungal community composition related to sample type (HTHT-nCp, HTHT-Cp, HTIT-nCp, and CTIT-Cp) was analyzed by two-dimensional nonmetric multidimensional scaling (NMDS) based on “Hellinger-transformed” abundance data on Bray-Curtis distance using the function “ordinate” (settings: trymax = 1,000) and plotted using the function “plot ordination” of the “phyloseq” package. Permutational multivariate analysis of variance (PERMANOVA) was performed to test differences in fungal community compositions between sample types, sampling location and tree age based on 999 permutations using the function “adonis” of the “vegan” package (Oksanen et al., 2019). Indicator taxa between healthy tissue of healthy trees (HTHT-nCp) and canker tissue of infected trees (CTIT-Cp) were analyzed on the genus level by linear discriminant analysis effect size (LEfSe) method. This statistical approach couples nonparametric tests and linear discriminant analysis (LDA) to identify taxa that significantly differ in relative abundance between the sample types, using the package “microeco” (Liu et al., 2021). The FUNGuild database (Nguyen et al., 2016) was used to classify OTUs into trophic modes (saprotroph, symbiotroph or pathotroph) and guilds (e.g., plant pathogen, wood saprotroph, etc.) within the “microeco” package. Alpha diversities between sample types (number of observed OTUs, Shannon, and Simpson indices) were calculated and tested for significant differences using the nonparametric Dunn’s Kruskal-Wallis test for multiple comparisons (KW_dunn option) of the “FSA” package (Ogle et al., 2022) and respective *p* value adjustment (method = “fdr”) implemented in the “microeco” package. Furthermore, this dataset was used to show the number of OTUs that were shared between sample types or exclusive to a respective group (“Venn diagram”). Finally, to ensure that differences in community composition between sample types are not only based solely on the presence or absence of *C. parasitica*, NMDS and PERMANOVA analyzes were also performed after excluding this species.

## Results

### Fungal community in European chestnut bark tissue

**Figure 1:**
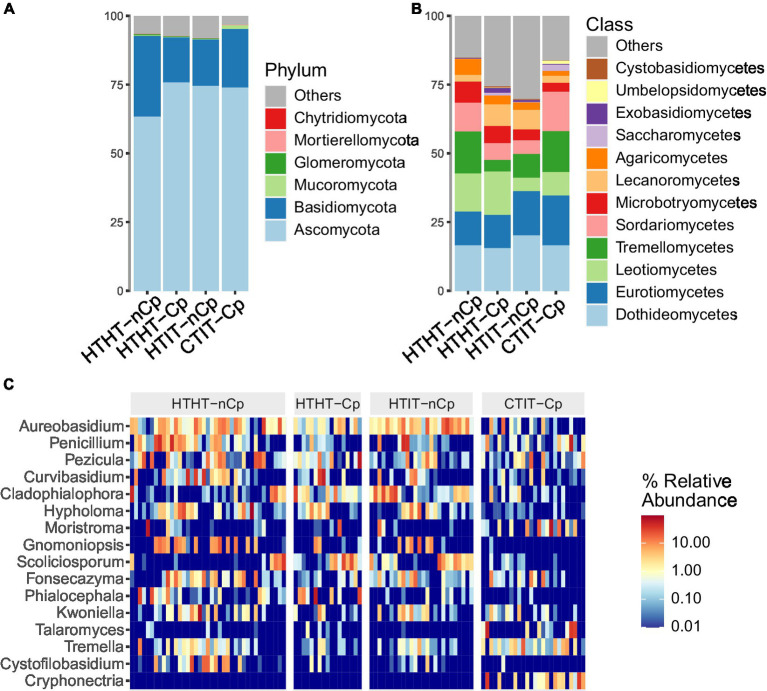
Taxonomic composition of fungal communities detected in HTHT-nCp (healthy tissue from healthy trees without *Cryphonectria parasitica*), HTHT-Cp (healthy tissue from healthy trees with latent *C. parasitica* infection), HTIT-nCp (healthy tissue from *C. parasitica* infected trees) and CTIT-Cp (canker tissue from *C. parasitica* infected trees). **(A)** Relative abundance at phylum level for each sample type; **(B)** Relative abundance at class level for each sample type, and **(C)** Heatmap showing relative abundance of the 16 most dominant genera for each individual sample and sample type.

The fungal community was assessed based on 116 samples, consisting of 39 HTHT-nCp (healthy tissue of asymptomatic trees without *C. parasitica*), 17 HTHT-Cp (healthy tissue of asymptomatic trees with *C. parasitica*), 39 HTIT-nCp (healthy tissue of infected trees), and 21 CTIT-Cp (cankered tissue of infected trees) samples. Illumina amplicon sequencing yielded a total of 8,589,604 raw sequences. Processing revealed 6,282,753 high-quality sequences, of which 5,231,246 sequences were classified as fungi and clustering into 2,365 OTUs. The rarefied dataset consisted of 108 samples, each comprising 5,543 sequences and 2,109 OTUs. The rarefaction curves (Supplementary Figure S2) reached a plateau, implying a saturated sequencing depth for the samples. Overall, the bark fungal community of European chestnut was highly dominated by the phyla *Ascomycota*, *Basidiomycota*, and *Mucoromycota*, accounting for 95% of all sequences (Figure 1A).

The *Ascomycota* was the most dominant phylum in all sample types, with 71% of sequences. *Basidiomycota* had higher abundance in HTHT-nCp samples (22%). *Mucoromycota* were detected at a higher abundance in the CTIT-Cp samples, to which the phylum *Chytridiomycota* was also restricted. The classes *Dothideomycetes*, *Eurotiomycetes*, *Leotiomycetes*, *Tremellomycetes*, *Sordariomycetes*, and *Microbotryomycetes*, in order of abundance, dominated in all sample types, with variable differences in relative abundance (RA) among the sample types (Figure 1B). The *Dothideomycetes* class was the most dominant in the *Ascomycota*, accounting for 18.6% of detected sequences. Within this class, the orders *Dothideales* and *Capnodiales* and the families *Aureobasidiaceae* and Cladosporiaceae dominated. *Tremellomycetes* was the most abundant class of *Basidiomycota*, where *Tremellales* dominated at the order level, and *Bulleraceae* and *Cryptococcaceae* dominated at the family level. The dominant genera identified were *Aureobasidium*, *Penicillium*, and *Curvibasidium* (Figure 1C), while the most taxonomically diverse genera were *Tremella*, *Cystobasidium*, and *Oidiodendron*. *Aureobasidium pullulans*, *Curvibasidium cygneicollum*, and *Penicillium raistrickii* were the most abundant species.

### Fungal community patterns in relation to *Cryphonectria parasitica* infection

**Figure 2:**
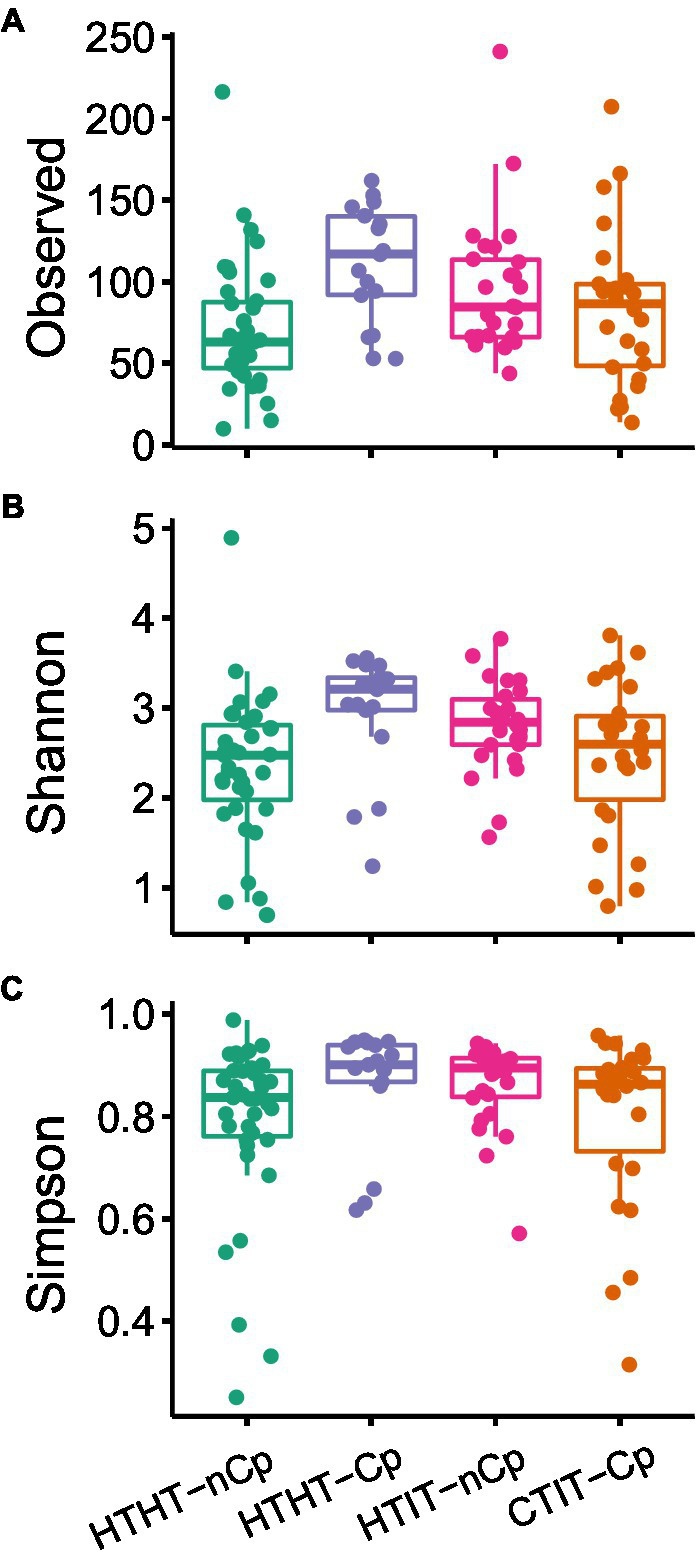
**(A)** Observed number of OTUs, **(B)** Shannon diversity, and **(C)** Simpson’s diversity index for fungal communities of HTHT-nCp (healthy tissue from healthy trees without *C. parasitica*), HTHT-Cp (healthy tissue from healthy trees with latent *C. parasitica* infection), HTIT-nCp (healthy tissue from *C. parasitica* infected trees) and CTIT-Cp (canker tissue from *C. parasitica* infected trees). Differences between the sample types were determined by the Dunn’s Kruskal–Wallis test for multiple comparisons, *p* < 0.05.

**Figure 3:**
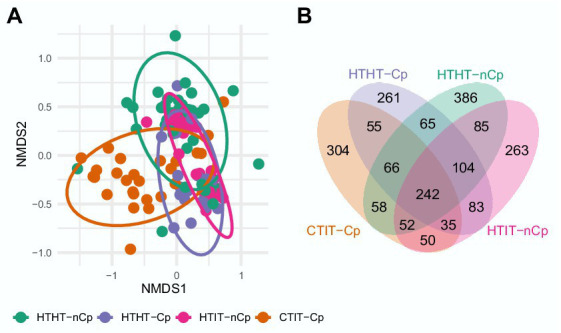
**(A)** Two-dimensional nonmetric multidimensional scaling (NMDS) plot of fungal communities in relation to sample type HTHT-nCp (healthy tissue from healthy trees without *C. parasitica*), HTHT-Cp (healthy tissue from healthy trees with latent *C. parasitica* infection), HTIT-nCp (healthy tissue from *C. parasitica* infected trees) and CTIT-Cp (canker tissue from *C. parasitica* infected trees); 2-D-stress value: 0.21. **(B)** Venn diagram showing shared and exclusive OTUs for the different sample types (HTHT-nCp, HTHT-Cp, HTIT-nCp, and CTIT-Cp) from bark tissues of European chestnut.

The α-diversity indices, including the number of observed OTUs, Shannon index, and Simpson’s diversity index, differed between sample types and showed the highest values for HTHT-CP and lowest for HTHT-nCp (Dunn’s Kruskal–Wallis test, *p* < 0.05; Figure 2). Nonmetric multidimensional scaling (NMDS) based on the Bray-Curtis distance metric revealed distinct fungal communities in relation to the sample types (Figure 3A). Based on fungal community composition, HTHT-nCp and the intermediate HTHT-Cp and HTIT-nCp samples clustered together, whereas CTIT-Cp samples tended to be distinctly separated.

PERMANOVA revealed significant differences between sample types, mainly attributed to the separation of fungal communities of CTIT-Cp, confirming the significant effect of canker formation (*F* = 4.2683, *p* = 0.001) in reshaping the fungal community composition (Figure 3; Supplementary Tables S1, S2). This was also evident in the Venn diagram (Figure 3B). Only 242 OTUs (i.e., 11.5%) of the 2,109 OTUs were detected in all sample types (Figure 3). An OTU affiliated with *A. pullulans* was present in 50% of the samples. The number of shared OTUs among individual samples was low, indicating lower ubiquity. The Venn diagram further showed relatively high similarity in the fungal communities between HTHT-nCp, HTHT-Cp, and HTIT-nCp (sharing 104 OTUs). The fungal assembly of CTIT-Cp was significantly dissimilar to that of all other sample types (sharing 50 to 58 OTUs with them). The PERMANOVA analysis showed that fungal community composition differed between juvenile and adult trees (*F* = 2.2218, *p* = 0.003) and between the two geographical locations (*F* = 3.4518, *p* = 0.001; Supplementary Table S2). All effects on fungal community composition were confirmed by reanalysis of the dataset after removing *C. parasitica* sequences (Supplementary Figure S3, Supplementary Table S3). Functional prediction *via* FUNGuild identified 3 trophic modes and 23 fungal guilds (Supplementary Figure S5) with no significant differences in the relative abundance between sample types, thus canker formation did not affect the ecological guild. Saprotrophic fungi represented the dominant trophic mode (more than 30% in all samples types) followed by pathotroph fungi (20–30%). Plant pathogen and fungal parasite represented the largest groups (10–18%), followed by wood saprotroph, animal pathogen and endophyte.

### Variation in taxonomical diversity in relation to *Cryphonectria parasitica* infection

**Figure 4:**
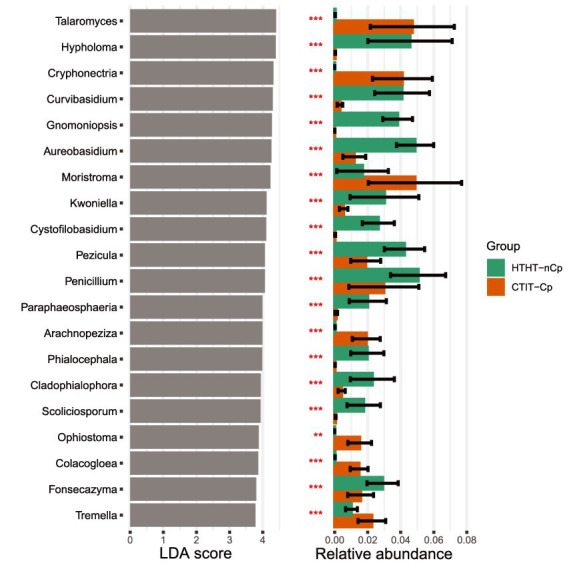
Top 20 indicator genera (*p* < 0.01) and their relative abundances for fungal communities inhabiting HTHT-nCp and CTIT-Cp samples from bark tissues of European chestnut identified using linear discriminant analysis (LDA).

LDA effect size (LEfSe) was applied to the HTHT-nCp and CTIT-Cp samples. LDA results revealed differentially abundant fungal genera, thus confirming the alteration in fungal abundance between HTHT-nCp and CTIT-Cp samples, although for some genera variability between samples was rather high (Figure 4). The genera *Cystofilobasidium*, *Gnomoniopsis* (with the unique species *G. smithogilvyi*), *Hypholoma*, *Phialocephala*, and *Scoliciosporum* were identified as indicator taxa for HTHT-nCp samples based on LEfSe, indicating a decreasing frequency or complete suppression of these taxa with respect to canker formation. Further indicator genera for HTHT-nCp were *Aureobasidium* (with unique species *A. pullulans*), *Penicillium*, *Pezicula*, and *Fonzecazyna*, for which the RA moderately decreased in the CTIT-Cp samples. *Arachnopeziza*, *Calacogloea*, *Cryphonectria* (with the unique species *C. parasitica*), *Moristroma*, *Ophiostoma*, *Talaromyces*, *Tremella, and Umbelopsis* were identified as indicator genera for CTIT-Cp samples, reflecting their enrichment in these samples. Importantly, in the CTIT-Cp samples, an increasing abundance of *C. parasitica* occurred simultaneously with a strong decrease in *G. smithogilvyi*. *Cryphonectria parasitica* was the most dominant species in canker samples, reaching up to 35% RA of sequences per sample. *Gnomoniopsis smithogilvyi* detected in 77% of the healthy samples, dominated the fungal communities of some individual samples with up to 43% sequence abundance. *Gnomoniopsis smithogilvyi* was detected in only 9% of the CTIT-Cp samples, and usually at approximately 1% RA of sequences (Figure 1C). *Cryphonectria parasitica* co-occurred latently with *G. smithogilvyi* in healthy tissue in which *C. parasitica* was detected at 1% RA of sequences.

## Discussion

This is the first study that employed metabarcoding to analyze changes in the fungal communities of chestnut trees with regard to canker formation. Previous studies have focused on the gradual change of the fungal communities in the chestnut canker by applying mostly culture-dependent approaches, which lack in resolution as they do not capture the total fungal diversity (Akilli et al., 2011; Kolp et al., 2018, 2020). In this study, we investigated and compared the fungal communities in healthy, *C. parasitica*-infected and canker samples to increase our understanding of how chestnut blight modifies fungal communities in the bark tissues of European chestnut.

The overall fungal community of European chestnut bark is composed of many phyla. *Ascomycota* dominated by far, followed by *Basidiomycota*, a pattern commonly detected in plant mycobiomes (Rodriguez et al., 2009; Bálint et al., 2015; Fort et al., 2016; Pellitier et al., 2019). *Ascomycota* species are generally known to have higher ecological plasticity and, thus can adapt better to difficult conditions than *Basidiomycota* (Lutzoni et al., 2004; Chen et al., 2017; Li et al., 2020). Basidiomycetes, except for basidiomycetous yeasts, have generally low abundance in healthy or senescing plant tissue as they tend to degrade more recalcitrant tissues at a later stage in microbial succession (Voříšková and Baldrian, 2013). Fungal communities of European chestnut bark were remarkably characterized by the abundance of the *Basidiomycota* yeast classes *Tremellomycetes* and *Microbotryomycetes*. Both classes are commonly reported in phyllosphere fungal communities (Bálint et al., 2015; Fort et al., 2016; Yao et al., 2019) and their members seem to adapt to changing and extreme environments as endophytes or saprobes (Sohlberg et al., 2015; Dresch et al., 2019). Fungi in these two classes showed high colonization of both healthy and canker tissues in European chestnut bark. The most abundant species were *Curvibasidium cygneicollum*, *Fonsecazyma betulae*, *Cystofilobasidium capitatum* and *Cryptococcus* spp.

As expected, our results revealed differences in fungal community diversity and composition according to sample types. Despite the variation in abundance of several taxa, fungal communities of the HTHT-nCp samples, representing the initial fungal communities, and those of the HTHT-Cp and HTIT-nCp samples representing the fungal communities at the early stage of *C. parasitica* infection, showed high similarity. Hence, we concluded that *C. parasitica* infection *per se* might have only a weak effect on the fungal community. The dissimilarity reported between fungal communities in the CTIT-Cp and non-canker samples (HTHT-nCp, HTHT-Cp, and HTIT-nCp) indicated that the canker formation was responsible for the difference in the fungal community. In our study, Shannon and Simpson diversity significantly decreased in the CTIT-Cp samples, thus denoting less diverse fungal community. As the fungal communities of these CTIT-Cp samples were largely dominated by *C. parasitica*, we hypothesized that the detection of further OTUs may be limited in such samples and the abundance of *C. parasitica* may influence that of all co-occurring fungi. This is likely to happen in the analysis of any mycobiome characterized by one predominant species when using high-throughput sequencing (Gloor et al., 2017). However, our results of NMDS and PERMANOVA obtained after discarding all *C. parasitica* sequences did not differ from those of the whole mycobiome dataset, thus confirming the canker impact on fungal community.

Another novelty of our study is that we started upstream from the fungal communities of healthy tissues to further follow variations in fungal communities in canker tissues of European chestnut, whereas Kolp et al. (2020) followed the temporal changes in fungal communities in American chestnut (*C. dentata*) canker tissues. Despite this, the outcomes of both studies are to some extent consistent or complementary with respect to fungal community changes in chestnut bark tissues infected with *C. parasitica*. Kolp et al. (2020) have shown that alterations in the American chestnut fungal communities occur as a dynamic process linked to the canker development stage. Our findings are consistent with those of Kolp et al. (2020), which further demonstrated that total fungal community decreased with increasing canker severity, corresponding to high frequency of *C. parasitica*. Kolp et al. (2020) also noticed that intense fungal recolonization occurred in cankers after receding of *C. parasitica*. Admittedly, the prevalence of *C. parasitica* decreases during canker recovering process so that the completely recovered canker may be free of *C. Cryphonectria*. However, based on this, it is not expected that the fungal communities in *C. parasitica*-free cankers can converge with that of healthy samples owing to the physicochemical changes in canker tissues. All canker samples in our study harbored *C. parasitica*, therefore we could not test this assumption, for instance by comparing fungal communities between the CTIT-nCp (canker tissue from infected trees without *C. parasitica*) and HTHT-nCp. This aspect needs to be addressed in any future study. Our data suggested some features that might underlie variation in species richness and composition of the fungal communities. In that respect, LEfSe analysis indicated both the establishment of new species and suppression of certain species in the CTIT-Cp samples.

The enrichment effects related to *C. parasitica* and canker formation agree with the findings of Russin and Shain (1984) that American chestnut tissues can be colonized by *Ceratocystis* spp. if they are previously infected by *C. parasitica*. Prospero and Rigling (2016) have shown that many other fungal species may actively colonize dead tissues necrotized by *C. parasitica* in cankers. Thus, it is plausible that the enrichment partly results from the nutrient accessibility in canker as the enriched species revealed in this study are all commonly wood-inhabiting saprotrophs. The functional role of enriched species in chestnut trees is currently unknown (Kolp et al., 2020). However, fungal communities in plant tissues influence disease dynamics (Partida-Martinez and Heil, 2011; Desprez-Loustau et al., 2016). Especially, some species that are usually abundant and co-occur with the pathogen may act as disease facilitators associated with higher severity of the disease (Busby et al., 2016). *Bulgaria inquinans* enriched in canker also occurs as endophyte and produces many antimicrobial bioactive metabolites (Li et al., 2013; Ariantari et al., 2019). Therefore, we estimate that fungal species enriched in infected samples as well as those with constant abundance deserve further investigation, as some may be potential biocontrol agents against chestnut blight.

In contrast, many abundant species in healthy samples, such as *G. smithogilvyi*, *Graphostroma platystoma*, *A. pullulans*, *Botryosphaeria stevensii*, *Penicillium bialowiezense*, and *Hypholoma fasciculare,* were rarely detected or completely absent in the CTIT-Cp samples. The possible suppressive effect on these species may reflect either the antagonism of *C. parasitica* and/or the canker microhabitat becoming unsuitable for their survival. Our results are in corroboration with those reported by Kolp et al. (2020, 2018) that *C. parasitica* has highly competitive ability toward co-occurring fungi in cankers. Similar competitive capacity has been reported for the brown-rot fungus *Fomitopsis pinicola*, which substantially reduces the richness of other wood-inhabiting fungi in spruce wood (Bässler et al., 2016). Competitiveness of *C. parasitica* might be a plausible hypothesis to explain the slight difference between the fungal communities of the HTHT-Cp and HTHT-nCP samples. In addition to antagonism, suppressive effect on the fungal community may be attributable to the changes, which evolve with the canker formation and likely benefit or disadvantage the growth of certain fungal species (McManus et al., 1989).

Canker formation causes the deterioration of the vascular cambium and the most recent growth ring in the xylem, and structural irregularities in vessels and fibers in the affected wood (Gunduz et al., 2016). *Cryphonectria parasitica* produces oxalic acid in the canker tissues, which contributes to its pathogenicity by lowering the pH and facilitating its degradative enzymatic action (Bateman and Beer, 1965; Mccarroll and Thor, 1978). Moreover, *C. parasitica* releases toxins and cell wall-degrading enzymes to kill host cells and access the nutrients (Havir and Anagnostakis, 1983). Other studies have shown the great adverse influence of the pH variation on the bark fungal colonization (Roane et al., 1986; Pellitier et al., 2019). Therefore, it cannot completely be ruled out that the decreased pH-value and altered chemical conditions may be growth inhibitors for fungi co-occurring with *C. parasitica*, hence affecting the fungal communities in European chestnut bark tissues. The distribution pattern of *G. smithogilvyi* reported herein is consistent with the findings of Kolp et al. (2020). They observed that *Gnomoniopsis* was more likely to be found in the margin of cankers than in canker center inhabited by *C. parasitica* at high relative abundance. Our study showed that *G. smithogilvyi* predominantly occurred as an endophyte and was detected in only a few *C. parasitica*-infected samples. Interestingly, these samples had very low relative abundance of *C. parasitica*, probably being at the earlier stage of infection or from canker margin. Hence, while both *C. parasitica* and *G. smithogilvyi* can cause canker on European chestnut, they may rarely coexist spatially.

It is worth mentioning that some species (i.e., *Trichoderma* and *Nectria*) commonly found at high abundance in chestnut bark fungal communities in studies relying on fungal culturing (Akilli et al., 2011; Kolp et al., 2020) were not detected in this study. This could due in part to the methodological difference. The strength of culture-independent method, such as amplicon sequencing used in this study, is the potential recovery of slow growing and unculturable species, but owing to biases related to ITS primers or barcode length (Mbareche et al., 2021), some easily isolated species may indeed go undetected (Kraková et al., 2017; Mendoza et al., 2017). Furthermore, climatic conditions such as summer rainfall and winter temperatures obviously affect chestnut blight and fungal community composition in cankers (Griffin et al., 1993), something that may cause local and temporal variation in mycobiomes. Therefore, it is noteworthy that the variation in canker fungal diversity recovered in this study and that of Kolp et al. (2020) is explained not only by the methodological difference, but also to some extend by the sampling schedule. We sampled toward the end of fall and Kolp et al. (2020) between summer and fall. Our results further suggested that also the plant community diversity at the sampled sites may affect chestnut bark fungal community. The proximity of oak stands may account for the detection of *Apiognomonia errabunda*, *Moristroma quercinum*, *B. stevensii*, and *Pezicula cinnamomea* on European chestnut, these fungi being frequently associated with oak diseases. Besides the host-jump occurring in mixed chestnut-oak stands and resulting in the settling of *C. parasitica* on oak (Dallavalle and Zambonelli, 1999; Radócz and Tarcali, 2009), little is known about the horizontal transmission between chestnut and oak associated fungi.

## Conclusion

This study demonstrated that the fungal communities of European chestnut bark are very diverse and are dominated by *Ascomycota* and *Basidiomycota*. Canker formation subsequent to *C. parasitica* infection leads to a decrease in fungal diversity and composition. Our study highlighted that *G. smithogilvyi* is mostly restricted to healthy chestnut trees at the study sites. These patterns suggest spatial exclusion of *C. parasitica* and *G. smithogilvyi* in European chestnut bark. However, the enrichment of many other species was observed in canker samples. In addition, based on our results, we conclude that amplicon sequencing, a useful tool for analyzing fungal community patterns, is adequate for the reliable identification of fungal pathogens in case of complex diseases and latent infection. Finally, we are aware that the recovered bark fungal communities of European chestnut and the differences observed among sample types in relation with canker formation may depend on the sampling time and location. Future amplicon-based studies combined with biochemical analyzes, extended seasonal and geographical sampling are required to further explore the drivers of fungal species composition and dynamics in European chestnut canker tissues and the functional roles of these fungi.

## Data availability statement

The datasets presented in this study can be found in online repositories. The names of the repository/repositories and accession number (s) can be found at: https://www.ebi.ac.uk/ena, PRJEB46887.

## Author contributions

CDM: conceived and designed the study and experiments, performed the experiments. JM and CDM: analyzed the data. CDM and JM: wrote the manuscript. All authors reviewed the manuscript. All authors contributed to the article and approved the submitted version.

## Conflict of interest

The authors declare that the research was conducted in the absence of any commercial or financial relationships that could be construed as a potential conflict of interest.

## Publisher’s note

All claims expressed in this article are solely those of the authors and do not necessarily represent those of their affiliated organizations, or those of the publisher, the editors and the reviewers. Any product that may be evaluated in this article, or claim that may be made by its manufacturer, is not guaranteed or endorsed by the publisher.
