# Supplementary material for: Bark-inhabiting fungal communities of European chestnut undergo substantial alteration by canker formation following chestnut blight infection
Source: Front Microbiol. 2023 Jan 26;14:1052031. doi: 10.3389/fmicb.2023.1052031 (PMC9911167; doi:10.3389/fmicb.2023.1052031)
Supplement: Supplementary file 1 [file Data_Sheet_1.docx]

Supplementary Material

Bark-inhabiting fungal communities of European chestnut undergo substantial alteration by canker formation following chestnut blight infection

Clovis Douanla-Meli^1*^, Julia Moll^2^

^1^Julius Kühn Institute (JKI) – Federal Research Centre for Cultivated Plants, Institute for National and International Plant Health, Messeweg 11-12, 38104 Braunschweig, Germany

^2^Helmholtz Centre for Environmental Research - UFZ, Department of Soil Ecology, Theodor-Lieser-Str. 4, 06120 Halle (Saale), Germany

*** Correspondence:**Corresponding Author Clovis Douanla-Meli, clovis.douanla-meli@julius-kuehn.de

**Figures S1-S5 and Tables S1-S3**


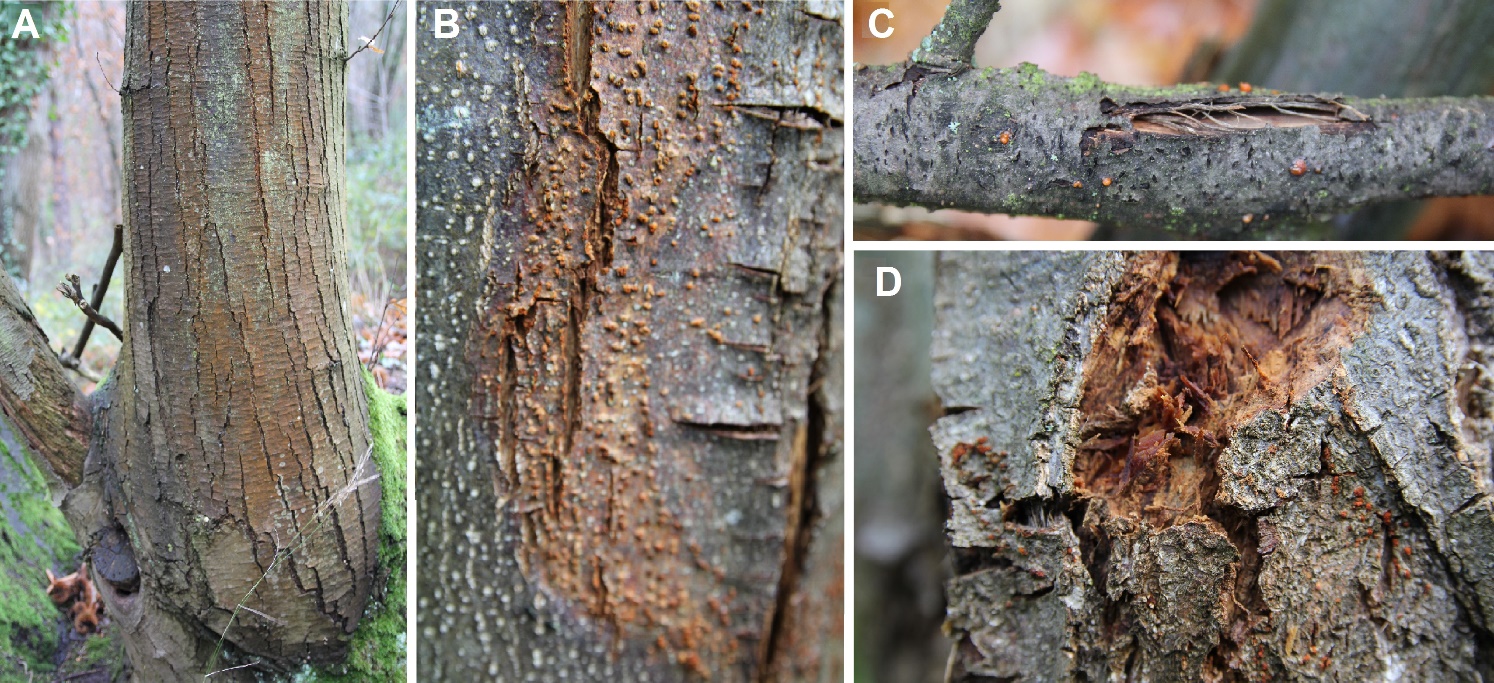


Supplementary Figure S1. *Castanea sativa* infected with *Cryphonectria parasitica*. A) Blushing and cracking of the bark. B-C) Cracked bark with numerous yellow-orange to red fruiting bodies. D) Canker or necrotic tissues (phloem and cambium) showing alteration.


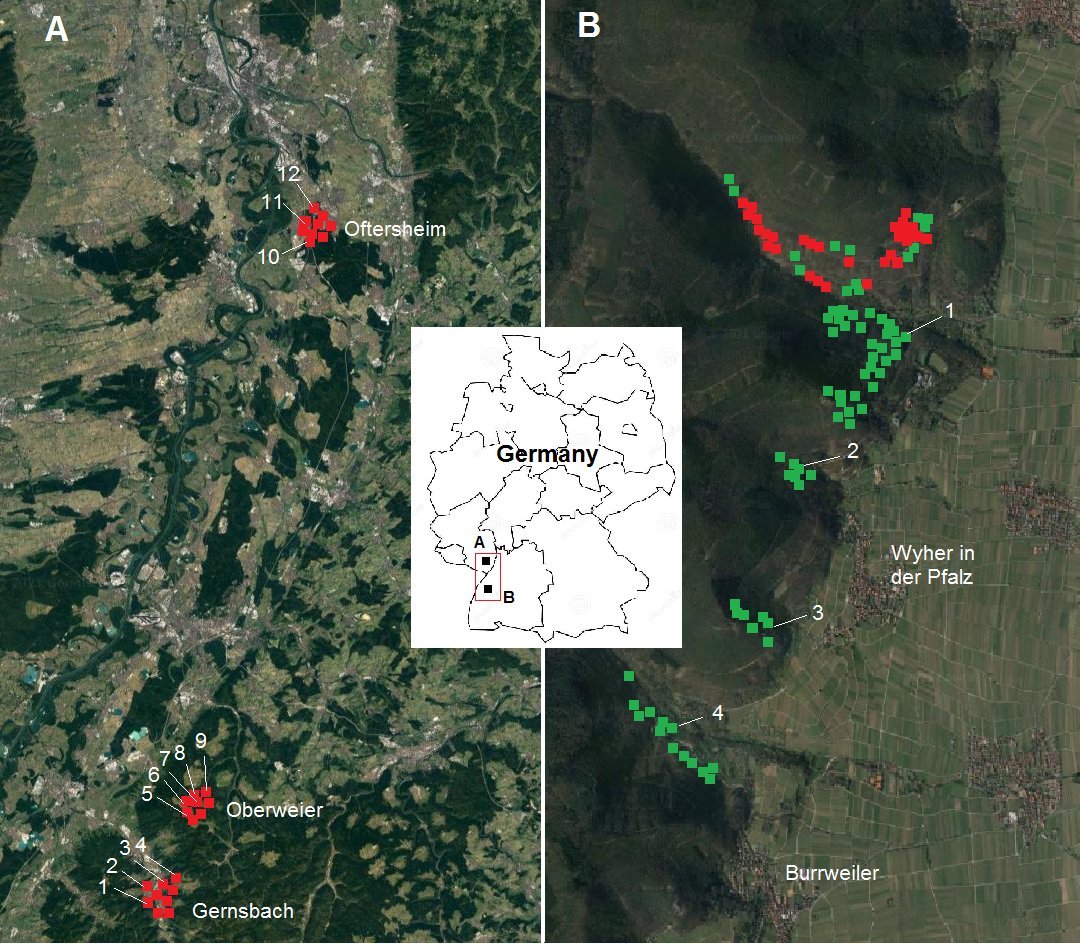


Supplementary Figure S2. Study sites. A) in Rhineland-Palatinate. B) in Baden-Württemberg. Green squares indicate sites free from chestnut blight (*C. parasitica*). Red squares indicate sites with chestnut blight infection.


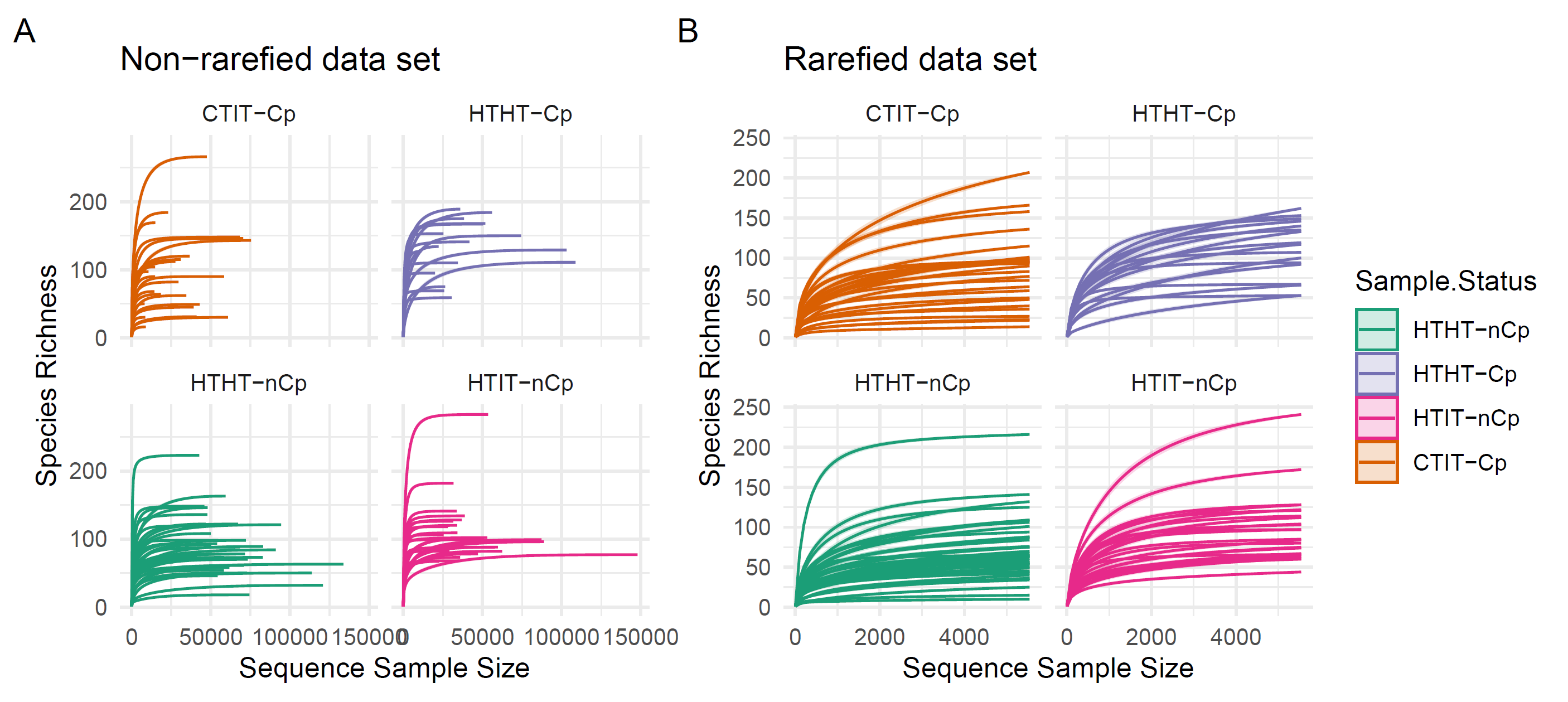


Supplementary Figure S3. Rarefaction curves for A) non-rarefied and B) rarefied data sets.


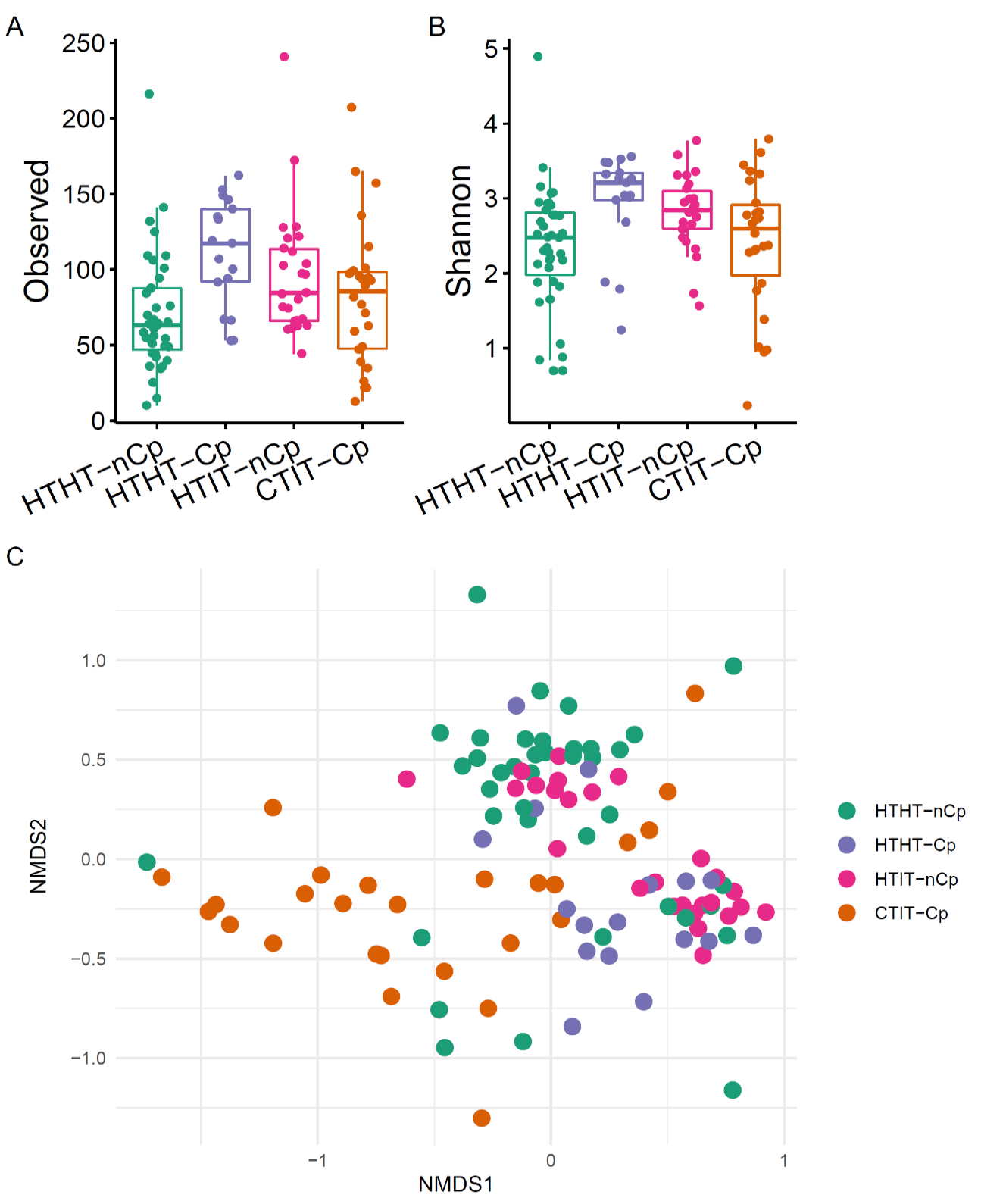


Supplementary Figure S4. A-B) Alpha diversity indices (observed and Shannon) for fungal communities richness and diversity in different sample types HTHT-nCp (n= 39), HTHT-Cp (n= 17), HTIT-nCp (n= 39) and CTIT-Cp (n= 41) from bark tissues of European chestnut based on the dataset excluding all sequences of *Cryphonectria parasitica*. Diﬀerences between groups were determined by the Kruskal-Wallis test, *p*< 0.05. C) Two-dimensional nonmetric multidimensional scaling (NMDS) plot based on Bray-Curtis distance displaying compositional variation in the fungal communities in relation to sample type (HTHT-nCp, HTHT-Cp, HTIT-nCp and CTIT-Cp); 2-D-stress value: 0.21. Analysis was based on dataset excluding sequences of *C. parasitica*.


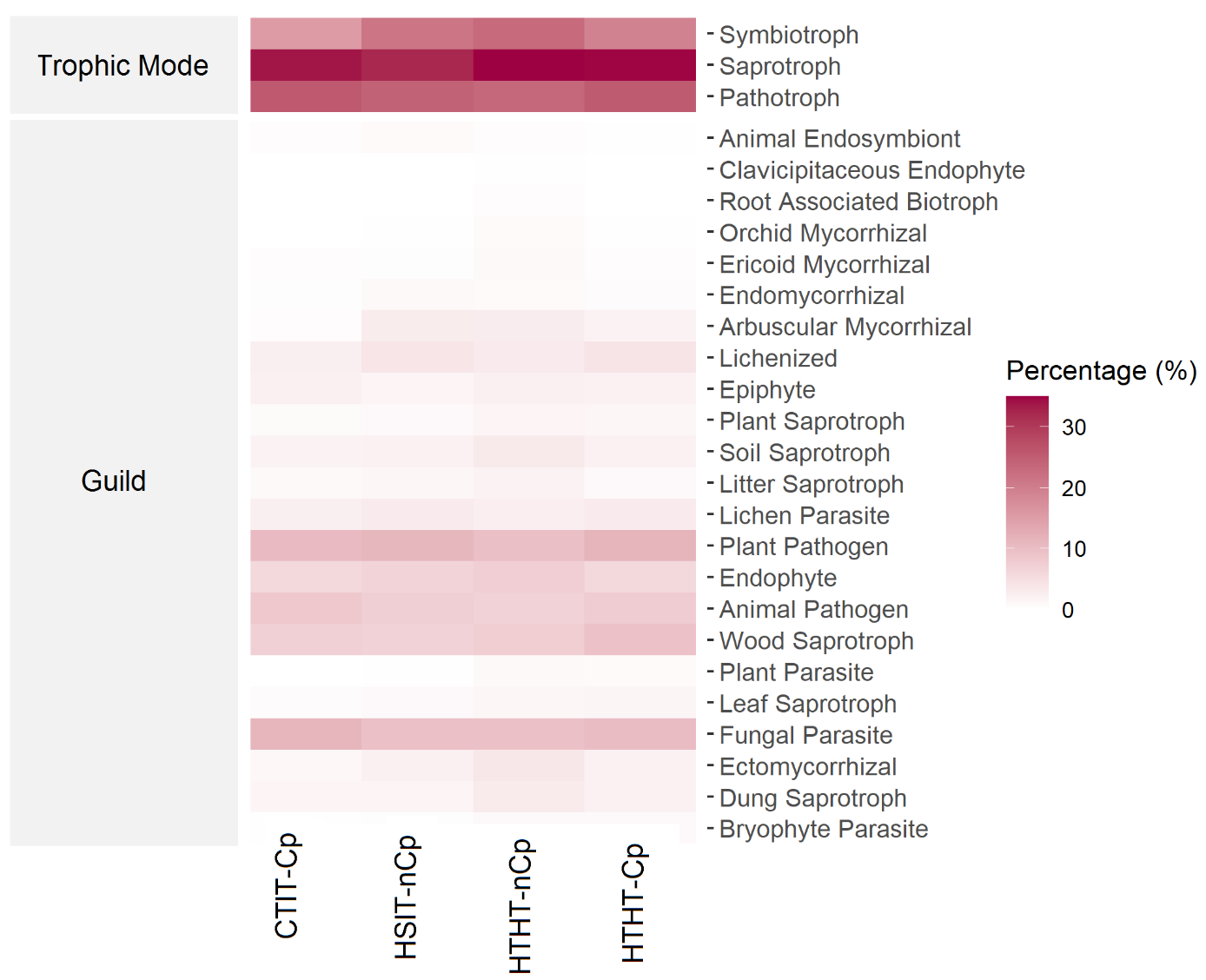


Supplementary Figure S5. Relative abundance of trophic modes (above) and functional guilds (below) of different sample types HTHT-nCp, HTHT-Cp, HTIT-nCp and CTIT-Cp from bark tissues of European chestnut.

**PERMANOVA results**

Supplementary table S1. Permanova results showing the effect of sample status (sample types HTHT-nCp, HTHT-Cp, HTIT-nCp and CTIT-Cp) on fungal community of European chestnut

|  | Df | SumsOfSqs | MeanSqs | F.Model | R2 | Pr(>F) |  |
| --- | --- | --- | --- | --- | --- | --- | --- |
| Sample status | 3 | 4.443 | 1.48101 | 4.171 | 0.10739 | 0.001 | *** |
| Residuals | 104 | 36.928 | 0.35508 | 0.89261 |  |  |  |
| Total | 107 | 41.371 | 1.00000 |  |  |  |  |

Signif. codes: 0 ‘***’ 0.001 ‘**’ 0.01 ‘*’ 0.05 ‘.’ 0.1 ‘ ’ 1

Supplementary table S2. Permanova results showing the effects of samples status (sample types HTHT-nCp, HTHT-Cp, HTIT-nCp and CTIT-Cp), geographical location (Baden-Württemberg, Rhineland-Palatinate) and tree age (juvenile, mature) on fungal community of European chestnut

|  | Df | SumOfSqs | R2 | F | Pr(>F) |  |
| --- | --- | --- | --- | --- | --- | --- |
| Sample status | 3 | 4.449 | 0.10628 | 4.2683 | 0.001 | *** |
| Region | 1 | 1.199 | 0.02865 | 3.4518 | 0.001 | *** |
| Tree age | 1 | 0.772 | 0.01844 | 2.2218 | 0.002 | ** |
| Residual | 102 | 35.438 | 0.84662 |  |  |  |
| Total | 107 | 41.858 | 1.00000 |  |  |  |

Supplementary table S3. Permanova results showing the effects of samples status (sample types HTHT-nCp, HTHT-Cp, HTIT-nCp and CTIT-Cp), geographical location (Baden-Württemberg, Rhineland-Palatinate) and tree age (juvenile, mature) on fungal community of European chestnut. SUBSET without sequences of *Cryphonectia parasitica*

|  | Df | SumOfSqs | R2 | F | Pr(>F) |  |
| --- | --- | --- | --- | --- | --- | --- |
| Sample Status | 3 | 4.328 | 0.10341 | 4.1382 | 0.001 | *** |
| Region | 1 | 1.188 | 0.02838 | 3.4073 | 0.001 | *** |
| Tree age | 1 | 0.776 | 0.01854 | 2.2257 | 0.002 | ** |
| Residual | 102 | 35.562 | 0.84966 |  |  |  |
| Total | 107 | 41.855 | 1.00000 |  |  |  |
